# Changes in Gut Phageome and Bacteriome Following Fecal Microbiota Transfer in Patients with Intestinal Graft-Versus-Host Disease and Crohn’s Disease

**DOI:** 10.3390/microorganisms13102337

**Published:** 2025-10-10

**Authors:** Alexei B. Chukhlovin, Oleg V. Goloshchapov, Oksana B. Shchukina, Aleksandra M. Kharitidis, Alexander A. Zhloba, Tatiana F. Subbotina, Aleksey V. Kusakin, Oleg V. Kosarev, Viktoria V. Tsai, Roman S. Kalinin, Yury A. Eismont, Oleg S. Glotov

**Affiliations:** 1 R.Gorbacheva Memorial Institute of Pediatric Oncology, Hematology and Transplantation, Pavlov First St. Petersburg State Medical University, 197022 St. Petersburg, Russia; golocht@yandex.ru (O.V.G.); burmao@gmail.com (O.B.S.); gastro-gepatocentr@mail.ru (A.M.K.); zhlobaaa@1spbgmu.ru (A.A.Z.); subbotina2002@mail.ru (T.F.S.); 2 Pediatric Research and Clinical Center for Infectious Diseases, 197022 St. Petersburg, Russia; axkusakin@gmail.com (A.V.K.); pancu43@gmail.com (R.S.K.); y-eis@inbox.ru (Y.A.E.); olglotov@mail.ru (O.S.G.); 3 Saint Petersburg Mining University, 199106 St. Petersburg, Russia; oleg.v.kosarev@yandex.ru; 4 Serbalab Laboratory, 199106 St. Petersburg, Russia; viktoriya14054@gmail.com

**Keywords:** fecal microbiota transplantation, virome, bacteriophages, Enterobacteriaceae, next-generation sequencing, plasma citrulline, graft-versus-host disease, Crohn’s disease

## Abstract

Intestinal bacterial dysbiosis develops in a number of immune-mediated disorders. Fecal microbiota transfer (FMT) is considered a potentially efficient tool for restoration of the patient’s gut microbiota. The aim of our study was to trace the time course of dominant bacterial populations and some *Enterobacteria* phages in patients with GVHD and Crohn’s disease after FMT procedure. Patients and methods: We observed 12 patients with intestinal graft-versus-host disease (GVHD), and 15 persons with Crohn’s disease after massive anti-infectious treatment. FMT was performed by a standard protocol using oral capsules administered for 2 days. Fecal bacteriome was assessed by 16S rRNA sequencing. Viral sequences were identified by NGS with a customized primer set. Plasma citrulline levels were measured in order to assess enterocyte damage in the patients. Results: Complete clinical response to FMT was observed in 5 of 12 GVHD patients and 10 of 15 Crohn’s disease cases. Before FMT, most anaerobic *Bacillota* were exhausted in both Crohn’s disease patients and GVHD. Following FMT, *Akkermansia* ratios tended to decrease within 30 days in Crohn’s disease, along with higher *Faecalibacteria, Romboutsia,* and *Dialister* ratios than in GVHD, thus suggesting lesser damage to anaerobic microbiota in Crohn’s disease. Increased contents of facultative anaerobes (*Enterococcus* and *E. coli*) was detected in GVHD patients after FMT. Fecal virome changes in Crohn’s disease after FMT included early transient decrease in *Caudoviricetes* with a rise in *Lederbergvirus* and *Eganvirus* ratios at later terms. In GVHD patients, reverse correlations were revealed between *E. coli* and *E. coli*-hosted *Eganvirus* species. Intestinal damage assessed by low plasma citrulline levels was associated with fecal *Klebsiella* expansion, being more pronounced in GVHD than in Crohn’s disease. Clinical response to FMT in GVHD patients correlated with increased plasma citrulline and lower *Eganvirus* abundance. Future studies will concern specific relations between fecal bacteriome and virome reconstitution following FMT in gut GVHD and other immune-mediated intestinal disorders.

## 1. Introduction

Intestinal microbiome contains multiple, mostly anaerobic, bacterial species. Severe gut bacterial dysbiosis may develop in various gastrointestinal disorders of autoimmune origin, such as intestinal form of acute graft-versus-host disease (GVHD), a severe immune-mediated complication of allogeneic hematopoietic stem cell transplantation (HSCT). Standard cytostatic therapy and antibacterial treatment in HSCT patients results in severe intestinal dysbiosis characterized by exhaustion of dominant bacteriome, [1]. Intensive cytostatic chemotherapy followed by allogeneic HSCT is often complicated by autoaggressive intestinal GVHD. Moreover, cytostatic treatment in these patients causes severe damage to intestinal mucosa causing bacterial translocation via gut barrier [2]. The enterocyte damage in HSCT and intestinal GVHD may be revealed by histology of mucosal biopsies, low citrulline levels in blood plasma [3].

Moreover, intestinal dysbiosis is observed in patients with Crohn’s disease, a chronic inflammatory bowel disorder of autoimmune genesis, which manifests with deep focal inflammatory lesions of large bowel [4]. Pronounced changes in gut bacterial and viral microbiota have been reported in these patients over the last years [5]. Alterations of bacteriome in Crohn’s disease may be caused by gene mutations in the human host, which modify the immune response to infectious agents [6,7]. Viral infections may also modify the clinical course of inflammatory bowel disorders [8]. In particular, some intestinal bacteriophages may provide anti-infectious action in Crohn’s disease [9].

Fecal microbiota transplantation (FMT) is considered a prospective but still experimental therapeutic option for recovery of normal microbiota in patients with pronounced gut dysbiosis, including patients with GVHD and with Crohn’s disease [1,10,11]. Recovery of gut bacteriome is considered a sufficient factor in FMT therapeutic effect. The viral component of intestinal microbiome is less studied. This numerous and variable community includes eukaryotic cell viruses (herpes group, adenoviruses, enteroviruses, etc.). Common reactivation of human viruses after HSCT is well studied, both in the blood and stool of the patients [12,13,14,15].

Gut virome contains myriads of bacteriophages, which are able to lyse their host bacteria (lytic forms), or coexist with them in symbiotic relations (temperate phages). Studies FMT as a treatment method of gut dysbiosis have suggested a sufficient role of phage populations in the FMT effects [16]. Transplantation of fecal microbiota or cell-free fecal transplants seems to be associated with engraftment of donor-derived bacteriophages, thus shaping bacteriome profile in the patients [17]. Hence, suppression and recovery of gut bacteriome suggests changing interactions between bacteriophage communities and their host bacteria. There are only several studies concerning changes in intestinal phageome following FMT in immune and infectious disorders [18]. A single case description reported activation of distinct viral populations following FMT in acute intestinal GVHD [19].

The aim of our study was to trace the time course of dominant bacterial populations and some *Enterobacteria* phages in patients with GVHD and Crohn’s disease. The patients were observed before FMT, and during follow-up for 60 days. Different patterns of intestinal dysbiosis were found in GVHD and Crohn’s disease at the pre-FMT step. *Enterobacteria*-infecting phages from *Eganvirus* genus showed inverse correlation with *E. coli*/*Shigella* dynamics post-FMT.

## 2. Materials and Methods

### 2.1. Ethical Statement

The study was conducted in accordance with guidelines of the 1964 Declaration of Helsinki and its later amendments. All techniques of stool sampling and collection for treatment used under this medical study were approved by the attending physician according to the institutional guidelines. All patients or their guardians signed a written informed consent to appropriate medical procedures, including written approval to FMT, analysis of stool microbiota, and potential usage of their clinical and laboratory data for clinical research. This study was approved by the Local Review Board of the Pavlov First State Medical University of St. Petersburg (ID number 214 of 17 December 2018).

### 2.2. Patients and Study Design

Our study included twelve adult patients (median age, 32 years) subjected to allogeneic HSCT, with primary diagnosis of leukemias (n = 9), aplastic anemia (n = 2), and myelodysplastic syndrome (n = 1). Clinical characteristics are shown in Supplemental Table S1. Allogeneic HSCT was performed, mainly from matched unrelated donors at the R.Gorbacheva Research Institute of Pediatric Oncology, Hematology and Transplantation (Pavlov University). The reduced-intensity conditioning regimens before allo-HSCT included fludarabine and busulfan. GVHD prophylaxis was performed with cyclophosphamide (Cy) on day +3, +4 after HSCT, followed by immunosuppression with either cyclosporine A, tacrolimus, mycophenolate, or ruxolitinib.

In most cases, allogeneic HSCT was accomplished by acute GVHD. Overall grading of GVHD was performed according to the EBMT Handbook (7th Edition) [20]. Intestinal GVHD was staged by clinical criteria [21]. The intestinal syndrome manifested with severe diarrhea and malnutrition. Current status of intestinal syndrome before and after FMT was assessed on Bristol scale (1 to 7 points) [22]. Abdominal pains were evaluated subjectively using a visual analogue scale. Mucosal inflammation was additionally confirmed by fecal calprotectin testing. The diagnosis of acute or overlap-type, steroid-resistant intestinal GVHD was also confirmed by intestinal histology, which showed lymphocyte infiltration of the epithelial wall, destruction and denudation of intestinal glands, and ulceration and local bleedings of large bowel mucosa corresponding to grade 4 GVHD [23].

The diagnosis of steroid-refractory acute GVHD was based on failure of preceding GVHD treatment, according to the Russian consensus, being in compliance with EBMT criteria [24]. Any conventional GVHD therapy (glucocorticosteroids, ruxolitinib treatment) failed in these patients, thus requiring FMT in order to restore normal gut microbiome.

During the HSCT procedure, all patients received prophylactic antibacterial therapy (mainly trimethoprim/sulfamethoxazole). In the early posttransplant terms, antibiotics were used in cases of febrile neutropenia, non-neutropenic fever, catheter-associated infections, sepsis, and other complications, as recently reported for a group of HSCT patients [25]. Upon development of acute GVHD, antibiotics were administered by clinical indications, being based on routine tests for antibiotic resistance of clinical isolates.

The comparison group consisted of 15 patients (9 males, 6 females) with clinically and histologically proven Crohn’s disease (median age, 30 years; 20 to 57 years old) admitted to the Department of General Medical Practice at the Pavlov University. The patients suffered severe colitis dependent on treatment with glucocorticosteroids and anti-inflammatory drugs (Supplemental Table S2). *C. difficile* infection was treated with systemic and topical therapy using vancomycin and metronidazole. The disease activity was assessed with the Harvey-Bradshaw index. Clinical decisions for FMT in Crohn’s disease were made in cases of poor response to the target therapy with cytostatics and monoclonal antibodies. The time period from *C. difficile* diagnosis to FMT was 110 (42–738) days.

A control (reference) group included 148 age- and gender-matched persons (9 to 53 years old) free of severe intestinal disorders who were subjected to 16S rRNA NGS studies of fecal bacteriome [26].

### 2.3. Pretransplant Examination of Microbiota Donors

Fecal microbiota transplants were performed from healthy donors. The ready-to-use biomaterial was provided from a commercial laboratory. Before donation, the candidate donors underwent thorough medical examination, and their feces were screened for basic microbiota bacteria and common enteric pathogens, according to standard protocol described elsewhere [27,28]. The fecal samples from donors were packed into plastic capsules and stored at −80 °C until their delivery to the clinic. The frozen encapsulated samples were given to the patients for oral uptake. In the GVHD group, FMT was performed at a median of 109 days (41–585) after HSCT, or at a median of 58 days (23–313) after the first signs of GVHD.

### 2.4. Fecal Microbiota Transfer

The FMT procedure was performed at the intensive care unit No. 3 of Pavlov University according to approved protocols based on previous FMT applications in *C. difficile* infection and GVHD [11,27]. In brief, the treatment included oral administration of 15 gelatine capsules with fecal microbiota (22 g/30 capsules) on two consecutive days. The stool samples for common bacteriology and NGS studies were collected within 3 days before FMT, and on days +1, +15, +30, +60 after the procedure.

### 2.5. Clinical Laboratory Studies

The intensive care patients were subjected to daily conventional assessment of blood counts, blood biochemistry, acute phase proteins (C-reactive protein, procalcitonin test). Common aerobic cultures of blood and fecal material were performed at the Microbiology Department of Pavlov University at the regular time points (pre-FMT to 60 days post-FMT), along with stool sampling for NGS studies. Diagnostic biopsies of rectal and ileal mucosa were performed and evaluated as elsewhere described [15].

To assess functional integrity of enterocytes in patients, we used a non-invasive method of plasma citrulline measurement by assessing amino acid profile in blood plasma samples taken at appropriate time points. The plasma samples were stored at −80 °C until analysis. Plasma amino acid profiles were determined in deproteinized samples by means of reversed phase HPLC [29], using Ultimate 3000, Thermo Scientific, USA, chromatograph with automatically precolumn derivatization by ortho-phthalic dialdehyde (OPA). Zorbax Eclipse AAA C18 column 4.6 × 150 mm; 3.5 µm (Santa Clara, CA, USA) was loaded with amino acids derivative by autosampler, eluted at 40 °C in 40 mM sodium phosphate buffer, pH 7.8, in a acetonitrile/methanol gradient. The analytic procedures were performed according to [25] and instructions from Agilent Technologies (Santa Clara, CA, USA). The citrulline concentration was expressed in μmol/L.

### 2.6. NGS Procedures

The entire procedure of DNA isolation from fecal lysates and preparation of 16S sequencing libraries was performed as previously described [28]. Briefly, the target fragments of the 16S rRNA gene were amplified with standard primers for the V3–V4 region. The purified PCR products were indexed with the KAPA HiFi HotStart ReadyMix (2X) (Roche Diagnostics, Switzerland) and Nextera XT Index Kit (Illumina, USA). The obtained libraries were sequenced using the Illumina MiSeq platform.

To detect the DNA sequences, we applied our original customized panel for targeted DNA sequencing of common eukaryotic viruses and bacteriophages [28] using the KAPA CustomPanel reagent set (Roche, Pleasanton, CA, USA). The aliquots of fecal DNA samples, being taken and treated in parallel, were subjected to 25 PCR cycles with KAPA HiFi HotStart ReadyMix (2X) (Roche Diagnostics, Zug, Switzerland). Indexing of the purified PCR products was also made as for 16S amplicons. The NGS procedure was performed by means of Illumina MiSeq platform.

### 2.7. Bioinformatic Analysis of the 16S rRNA NGS Data

Quality control of the sequencing reads was performed with FastQC software (version 0.11.9) [30]. Trimmomatic (v0.39) software was used for subsequent filtering and adapter removal [31]. The microbial composition of the samples was analyzed using the VSEARCH software (version v2.22.1) [32]. Chimeric sequences were removed using reference-guided chimera detection with the RDP “Gold” database, retaining non-chimeric sequences. To classify the bacteria and viruses, we used operational taxonomic units (OTUs). This approach was driven by a desire to combine our recent results with initially obtained data. To identify OTUs from the 16S sequencing database, we used the pipeline described in [28]. Bacterial taxonomy was derived from the generated OTUs by alignment with data from Ribosomal Database Project (RDP) V16 database [33]. The cutoff for taxonomy assignment was set at 0.7, and the output included the OTU ID and the taxonomic lineage.

Viral sequences revealed by NGS were analyzed by means of Kaiju bioinformatics software [34], version 1.10.1, in order to provide taxonomic identification and abundance estimation. In addition, the ‘—minimum-hit-groups 3’ parameter was used to set the minimum number of hit groups equal to 3 that must be found to declare the sequence classified. The Pavian (v1.2.1) tool was used to visualize the obtained data and produce tables for different taxa [35].

The raw NGS data have been deposited with links to BioProject accession number PRJNA1337300 in the NCBI BioProject database (https://www.ncbi.nlm.nih.gov/bioproject/) (accessed on 10 August 2025).

The final results of all samples were summarized in a single table [36]. The abundance tables for different taxa were generated with R package (phyloseq v1.44.0) [37]. Alpha and beta diversity values were obtained using the ‘estimate_richness’ and ‘distance’ functions of the phyloseq package release 3.21. The ‘heatmap’ function was used to plot the heatmap graph.

The inter-group differences for bacterial and viral taxa were evaluated by parametric methods (*t*-test) and by means of non-parametric tests. Spearman correlation analysis was performed to evaluate pairwise associations between viral and bacterial abundances. The resulting correlation matrix was visualized as a hierarchically clustered heatmap using Euclidean distance for both row and column clustering. All statistical analyses were conducted in R v.4.3.2 (R Core Team, 2023), with heatmap generation implemented through the ComplexHeatmap, package [38], version 2.13.1.

## 3. Results

### 3.1. Clinical Effects of FMT in GVHD and Crohn’s Disease

Complete clinical response after fecal microbiorta transfer was registered in six GVHD cases out of 12 (Supplemental Table S1), being characterized by stool and water balance normalization, relief of pain syndrome, and fever. Four patients exhibited only partial effect in terms of stool quality and pain syndrome. No clinical response to FMT was observed in two cases.

In the Crohn’s disease group (n = 15), 10 patients achieved clinical remission within 60 days following FMT, according to the Harvey-Bradshaw index of the disease activity (Supplemental Table S2). Four patients developed only moderate response, and absence of clinical response was noted in one case.

**Figure 1:**
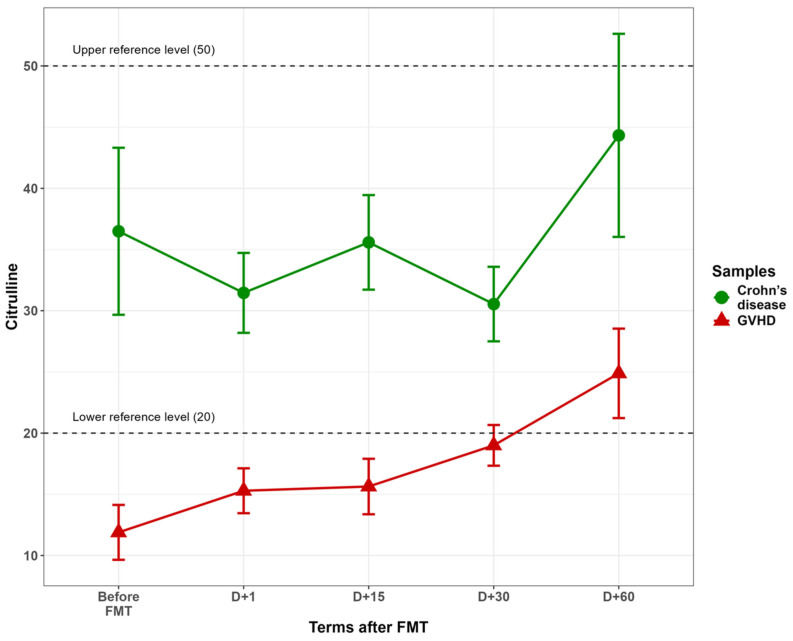
Plasma citrulline levels in blood plasma before and after fecal microbiota transfer in the patients with aGvHD (red triangles) and in Crohn’s disease (green circles). Abscissa, terms after FMT (days), ordinate, citrulline concentrations, µmol/L.

Low citrulline level in blood plasma was used as a well-known quantitative marker of enterocyte damage. The initial (pre-FMT) levels of plasma citrulline proved to be much lower in GVHD patients than in Crohn’s disease (11.9 ± 2.2 vs. 36.49 ± 6.8 µmol/L; *p* = 0.0005). Mean values of this index in GVHD up to 30 days after FMT were under reference levels for adults (20 to 50 µmol/L) [39]. The reduced citrulline levels in GVHD corresponded to clinical intestinal syndrome with extensive damage confirmed by mucosal biopsies (diffuse denudation and ulceration of intestinal cell layers). The average plasma citrulline levels in GVHD tended to increase over time, reaching subnormal levels by the day 60 after FMT, along with clinical improvement in some GVHD patients (Figure 1). In Crohn’s disease, plasma citrulline was at normal or high levels both before and after FMT, thus confirming more local lesions of intestinal mucosa in this disorder.

**Figure 2:**
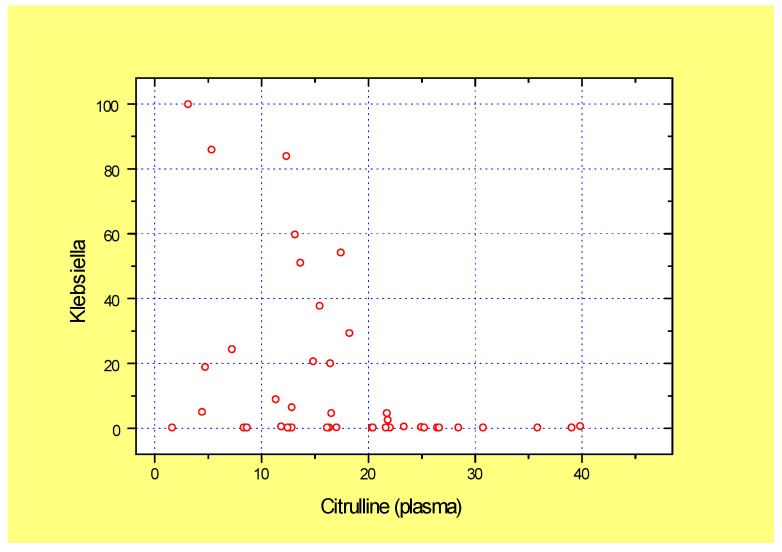
Correlation between low plasma citrulline levels (abscissa) and fecal *Klebsiella* abundance (ordinate) in total group of GVHD patients, both pre- and post-FMT (r = −0.395; *p* = 0.004).

Worthy of note, the low plasma citrulline index was associated with high abundance of potentially pathogenic *Klebsiella* in fecal samples of GVHD patients, thus suggesting clinical relevance of low plasma citrulline as a biomarker of enterocyte damage (Figure 2). Similar reverse correlation was detected with *Pseudomonas* abundance (r = −0.436; *p*= 0.002; n = 44).

### 3.2. Relative Contents of Fecal Bacteriome Before and After FMT

#### 3.2.1. Ratios of Bacterial Genera Before FMT

To determine initial pre-FMT characteristics of bacteriome associated with intestinal syndrome in GVHD and Crohn’s disease, we have assessed relative abundances of various bacterial genera in GVHD and Crohn’s disease by means of 16S rRNA sequencing. The relative contents of major bacterial genera (>1% in either of the three study groups) were compared to the samples from reference group (Table 1), The comparisons between GVHD and Crohn’s disease showed an increase in *Enterococcus* ratio in GVHD patients combined with decrease in potentially probiotic *Bifidobacterium*. A significant deficiency of basic anaerobic bacteria (especially, *Bacteroides*, *Blautia*, *Lactobacillus Parabacteroides*, *Roseburia*, *Ruminococcus*, *Suterella*) is revealed in both groups of patients when comparing with reference group. Moreover, an increase in potential pathogens (*Enterococcus*, *Klebsiella*, *Staphylococcus*) was detected in GVHD patients vs. the reference group.

#### 3.2.2. Bacteriome in Crohn’s Disease After FMT

**Figure 3:**
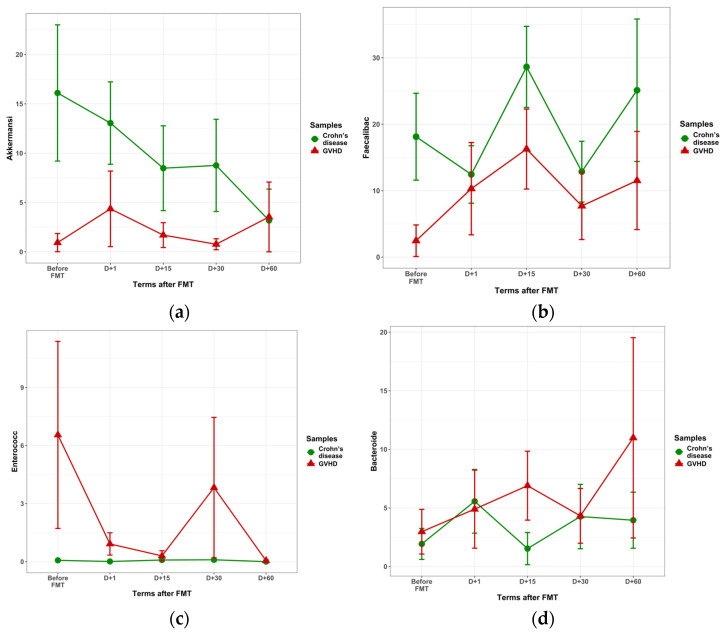
Relative contents of some dominant bacterial genera detected by 16S rRNA NGS in Crohn’s disease (gree circles) and GVHD patients (red triangles). Abscissa, timepoints of fecal sampling, days. Ordinate, Relative abundance (% of total counts) of dominating bacterial genera (>5% of total bacterial contents in each group). Denoted are: (**a**) *Akkermansia*; (**b**) *Faecalibacterium*; (**c**) *Enterococcus*; (**d**) *Bacteroides*.

The main changes in fecal bacteriome following FMT are shown in Figure 3. In particular, the time-dependent changes in some bacterial genera were different in Crohn’s disease vs. GVHD. In Crohn’s disease, the pre-FMT *Akkermansia* contents were higher than in GVHD, tending to decrease by 30 days after FMT, as seen in Figure 3a (*p* = 0.09). The mean ratios of *Faecalibacteria* proved to be higher in Crohn’s disease (n = 69) vs. GVHD (n = 51) at all time points after FMT (*p* = 0.009), as shown in Figure 3b. *Enterococci* were nearly absent in fecal microbiota in Crohn’s disease, both pre- and post-FMT. (Figure 3c).

Of other findings, relative amounts of *Romboutsia* in Crohn’s disease n = 32) were increased vs. GVHD (n = 23) on days 1 and 15 post-FMT (*p* = 2 × 10^4^. Higher *Dialister* contents in Crohn’s disease (n = 27) against GVHD (n = 19) were registered on days 15 and 30 following FMT (*p* = 0.011). Hence, the ratios of *Akkermansia* and *Faecalibacteria* in Crohn’s disease were increased during the 60-day observation period, *Romboutsia* and *Dialister* increased post-FMT in Crohn’s disease at different timepoints after FMT, whereas *Bacteroides* and *Enterococcus* showed a decrease under reference levels.

#### 3.2.3. Bacteriome Changes in GVHD Following FMT

In GVHD patients, *Akkermansia* levels remained low at all time points after FMT (Figure 3a). *Faecalbacterium* genus did not show a significant increase with time in both groups (Figure 3b). Abundance of *Enterococcus* was steadily increased in GVHD at different terms after FMT in contrast to near-zero *Enterococcus* contents in Crohn’s disease (Figure 3c; *p* = 0.005). Likewise, a low ratio of *Bacteroides* group was observed in both disorders (Figure 3d). However, by 15 days after FMT, we observed an increased *Bacteroides* ratio in GVHD (n = 12) over Crohn’s disease (n = 16; *p* = 0.048) without significant differences at other terms. High abundance of *Klebsiella*, a potential pathogen, was found in both groups of patients before FMT (Table 1) being also observed after FMT (*p* = 0.03), thus confirming our earlier results in limited groups [28]. *Bacteroides* were revealed at low levels during the whole period after FMT (Figure 3d).

### 3.3. Fecal Virome in the Course of FMT

#### 3.3.1. Pre-FMT Diversity of Viral Populations

We have performed a pilot study of fecal virome using a customized primer set detecting some clinically sound viral sequences. The study of 105 fecal samples revealed mostly bacteriophages from the *Caudoviricetes* order (over 85% of total), with only occasional findings of human viruses (Cytomegalovirus, CMV, and Adenovirus, AdV) in single patients with Crohn’s disease.

The incidence of distinct bacteriophage genera before FMT did not differ between GvHD and Crohn’s disease patients, as seen in Table 2. The only exception was a marginal increase in *Traversvirus* abundance in Crohn’s disease patients. *Eganvirus*, *Lederbergvirus,* and *Tequatrovirus* proved to be dominant phage genera comprising about 50% of total phage population detected by our targeted NGS panel.

Biodiversity of the viral species included *Caudoviricetes*. When comparing viral biodiversity before FMT, one may see near-similar α-biodiversity index in both disorders (Table 3). Meanwhile, the Chao 1 index in GVHD tended to be higher than in Crohn’s disease patients (*p* = 0.085).

**Figure 4:**
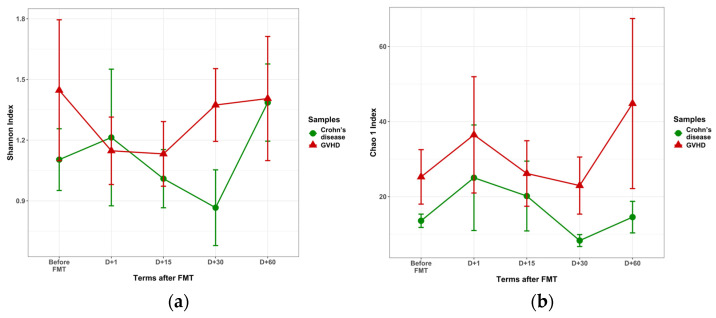
Time course of Shannon (**a**) and Chao 1 (**b**) biodiversity indexes for the fecal viruses in GVHD and Crohn’s disease following FMT in GVHD (red triangles) and Crohn’s disease patients (green circles).

The α-diversity of viral genera post-FMT (Shannon index) was similar in both GVHD and Crohn’s disease. Meanwhile, the Chao1 index proved to be higher in GVHD at all time points (*p* = 0.049), as shown in Figure 4. This finding may be caused by significant enrichment in *Enterobacteria* phages which are mostly presented in our NGS panel.

#### 3.3.2. Post-FMT Changes in Fecal Virome

**Figure 5:**
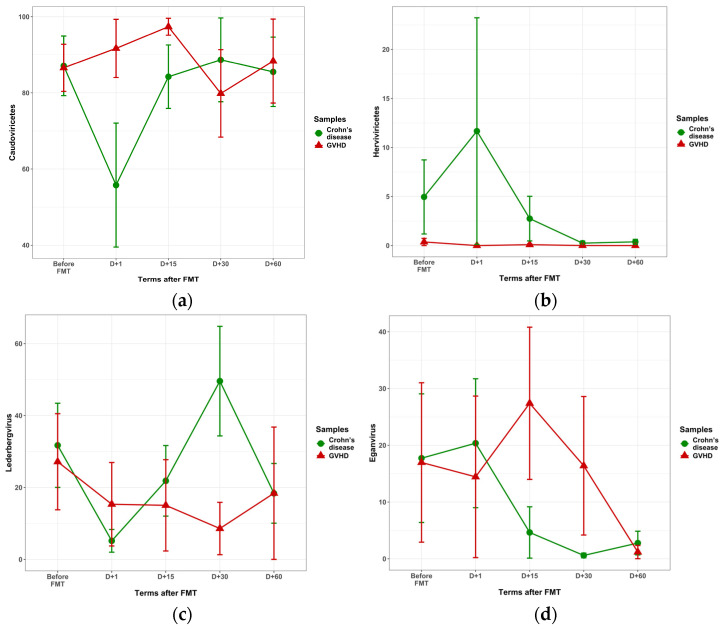
Relative contents of Caudoviricetes (**a**), Herviviricetes (**b**), *Lederbergvirus* (**c**), and *Eganvirus* (**d**) at different terms after FMT in GVHD (red triangles) and Crohn’s disease (green circles).

Abundance of *Caudoviricetes* was similar in GVHD and Crohn’s disease before FMT, (Figure 5a). The significant decrease in *Caudoviricetes* was found on day 1 post-FMT in Crohn’s disease versus GVHD (55.8 vs. 91.7%; *p* = 0.03), along with a non-significant increase in *Herviviricetes* (11.7% versus 0; *p* = 0.17). At the genera level, we revealed higher *Lederbergvirus* ratios in Crohn’s disease by the day 30 (49.6 + 15.3% vs. 8.7 + 7.3%, *p* = 0.03) as seen in Figure 5c. The relative *Eganvirus* content in Crohn’s disease was decreased on days 15 and 30 (2.8 + 2.5 vs. 21.5 + 8.8%, *p* = 0.012), as shown in Figure 5c. For other viral genera shown in Table 2, no significant changes were found.

### 3.4. Potential Interactions Between Fecal Bacteria and Phages

#### 3.4.1. Differential Clustering of Fecal Bacteria and Phages

**Figure 6:**
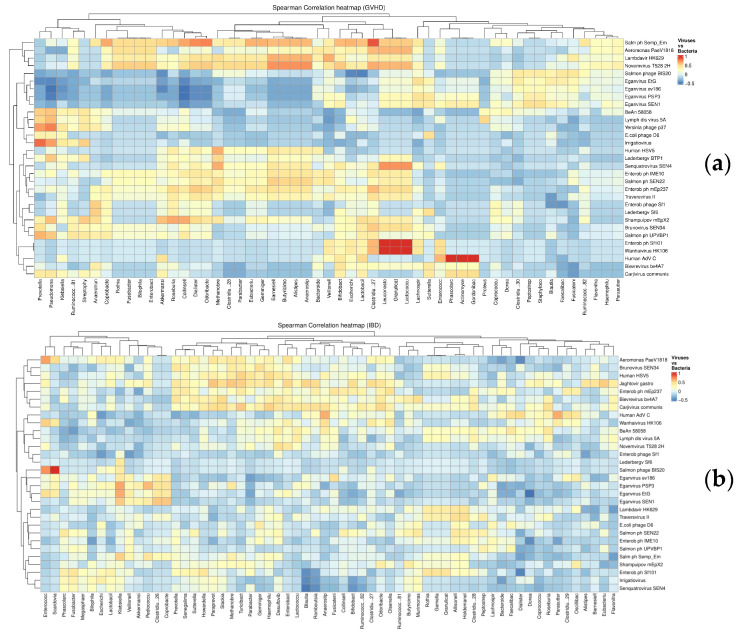
Correlation matrix between bacterial genera (horizontal axis) and viral genera (vertical axis) in GVHD (**a**) and Crohn’s disease patients (**b**).

General distribution of bacterial and viral genera was obviously different for both studied clinical groups. In GVHD samples (Figure 6a), we looked for negative interactions between bacteriophages and their hosting bacteria. The correlation matrices show a cluster of strong negative correlations (shown in deep-blue) between a group of *Eganvirus* and several potentially pathogenic bacteria (*Pseudomonas*, *Klebsiella*, *Prevotella*), as well as with *Akkermansia*, *Lactobacillus,* and *E. coli*. Of special significance is a negative correlation found between *Eganvirus* and *E. coli*/*Shigella* in GVHD patients (r = −0.570; *p* = 0.0004).

Such a pattern is not observed in Crohn’s disease patients (Figure 6b), with highly scattered associations, for example, between *Blautia* and SEN4 bacteriophage, or *Dialister* and *Dorea* with some *Enterobacteria*-hosted phages in the right part and the bottom of the diagram. These correlations of distinct phages with minor groups of anaerobic bacteria may be casual events, and should be later tested with larger samples.

#### 3.4.2. Correlations Between Phage Genera and Their Host Bacteria

In the total group of FMT-treated patients, a weak negative correlation was revealed between relative contents of *E. coli*/*Shigella*, and *Eganvirus* (−0.274; *p* = 0.008; n = 76).

Therefore, possible interrelations between the *Caudoviricetes* abundance and their host bacteria have been searched for in more detail for 26 detectable phage species, which are known to be hosted by Enterobacteria. Due to high scatter of different viral genera across the sample series, we have grouped together the phages for their hosting by *E. coli* (n = 12 species), *Enterobacteria* (n = 6 species), and *Salmonella* (8 species), as presented in NCBI Taxonomy Browser (https://www.ncbi.nlm.nih.gov/Taxonomy/Browser/wwwtax.cgi), (accessed on 4 June 2025).

Among the bacteriophages detected by our NGS panel, several *E. coli*-hosted phages (mostly, from *Eganvirus* genus) showed reverse correlations with *E. coli*/*Shigella* contents in fecal samples (Table 4). This negative correlation between contents of *E. coli* and Eganviruses was highly significant in GVHD group (r = −0.570; *p* = 0.004). In Crohn’s disease, neither of the 26 studied phages, including *Eganviruses*, showed significant correlations with *E. coli* contents (Table 4).

**Figure 7:**
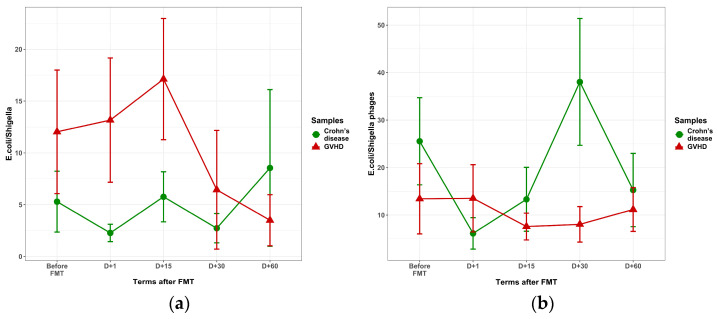
Time course of *E. coli*/*Shigella* (**a**) and *E. coli*/*Shigella*-hosted (**b**) bacteriophages contents in fecal samples after FMT in patients with GVHD (red triangles) versus Crohn’s disease (IBD, green circles). Abscissa, terms after FMT, days; ordinate, relative contents of *Enterobacteria* phages in stool samples.

The post-FMT time dynamics of fecal *E. coli* and *E. coli*-hosted phages are shown in Figure 7. In GVHD patients, the mean *E. coli* levels showed a trend for increase before and within 15 days after FMT (Figure 7a), along with steadily low levels of *E. coli*-hosted phages (Figure 7b). In Crohn’s disease, low *E. coli* were observed, with *E. coli* phages showing tending to increase by day 30 after FMT (38% vs. 8%, *p* = 0.207).

Another finding is an association between the clinical effect of FMT, and *Eganvirus* abundance in the samples (n = 39) from GVHD patients (8.9 ± 4.9% in complete response versus 25.8 ± 9.8% in partial or failed response; *p* = 0.002). Such dependence was not revealed in Crohn’s disease patients, thus confirming a significant role of *Eganvirus* for clinical effect of FMT in GVHD patients.

## 4. Discussion

### 4.1. Intestinal Damage and Plasma Citrulline in GVHD

GVHD grade and organ involvement in allotransplant patients was assessed by common clinical criteria [20]. More recently, extensive studies from the Mount Sinai Consortium (MAGIC) have been summarized in advanced GVHD standardization protocol employing GVHD-specific biomarkers for prognostic purposes [40,41]. One should note that clinical diagnostic approaches to GVHD grading have shown high correlation with the MAGIC-based approach [42].

Low plasma citrulline content is a well-known quantitative marker of enterocyte loss in post-HSCT patients [3]. Reduced plasma citrulline in these patients is considered a predictor of acute GVHD [43]. In our GVHD group, deeply decreased citrulline levels were also found, thus presuming extensive enterocyte loss. The citrulline levels yet reached subnormal levels by 60 days after FMT, being an indirect sign of intestinal mucosa recovery.

Unlike intestinal GVHD, Crohn’s disease manifests with deeper but more segmental lesions of terminal colon [44]. In Crohn’s disease, the citrulline plasma contents remained at high levels both before and after FMT, thus confirming less diffuse lesions of intestinal epithelium in this disorder.

### 4.2. FMT-Associated Bacteriome Changes

We have performed a comparative study of bacteriome and virome changes before and after FMT in patients with severe intestinal GVHD and Crohn’s disease. Both conditions present with immune-mediated inflammation of digestive tract and microbial dysbiosis [45].

As seen from Table 1, low pre-FMT abundance of anaerobic fecal bacteria, such as *Blautia, Faecalibacterium, Lachnospiracea* in both GVHD and Crohn’s disease, along with well-known domination of *Klebsiella* as reported in our earlier work [28]. These results confirm previous data on anaerobic bacteria exhaustion and higher prevalence of potentially pathogenic *Enterobacteria* after massive antibiotic treatment in HSCT patients [46]. In our Crohn’s disease group, antibiotic therapy was also applied in most cases (Supplemental Table S2).

In pre-FMT patients with GVHD, along with exhaustion of strict anaerobes (*Akkermansia, Faecalibacterium*), we found high *Enterococcus* abundance. This type of dysbiosis is well known, being a predictor of poor outcome among HCT patients [47].

We revealed initially low *Bacteroides* levels which may be explained by massive antibiotic treatment, both in GVHD and Crohn’s disease. After FMT, the reduced *Bacteroides* abundance was retained, except for a transient increase in GVHD patients which may be ascribed to positive clinical effect of FMT. Some authors consider *Bacteroides* a prospective next-generation probiotic for treatment of intestinal dysbiosis [48].

In Crohn’s disease, an initial increase in *Akkermansia* contents was noted, which tended to decrease with time after FMT, along with rise in *E. coli*/*Shigella* (*Enterobacteriaceae* family) in later terms. This finding suggests a probable disease remission of Crohn’s disease [49]. An increased *Dialister* abundance in pediatric Crohn’s disease also seems to be associated with clinical remission [50].

### 4.3. Phageome Changes After FMT

A variety of viruses infecting human eukaryotic cells are detected in clinical laboratories by means of multiplex PCR panels which, however, cannot cover the numerous viral agents in complex biological samples. The multiple-target NGS platforms may be effective for studies of certain viral genera. Such a broad-range approach may apply to NGS panels, specific for certain classes of eukaryotic viruses. Jansen et al. (2020) studied human fecal samples of hematopoietic stem cell recipients by means of NGS-based ViroCap test system using large array of DNA probes targeted for 34 families (337 species) of eukaryotic viruses [14]. The study enabled detection of different human viruses at higher sensitivity levels compared to conventional PCR tests, thus allowing a search for novel associations between viral infection and intestinal GVHD.

Meanwhile, the whole-genome metagenomic approach may reveal a quite broad spectrum of viruses and virus-like particles including bacteriophages of potential clinical significance [51]. Earlier metagenomic studies in 44 HSCT patients using NGS HiSeq techniques have revealed a “bloom” of eukaryotic cell viruses during the acute cytopenic period, e.g., herpes, polyomaviruses, papillomaviruses, and picobirnavirus, which could be a modifying factor of intestinal GVHD [12]. The abundance of fecal bacteriophages (except of *Siphoviridae*) was decreased, probably due to exhaustion of bacterial microbiome after intensive antibiotic therapy of HSCT patients.

Our targeted NGS viral panel allowed us to detect both human viruses and dominating phages infecting mostly *Proteobacteria*. By definition, such panel covers limited categories of target genes, thus allowing us to trace distinct phage genera in time- and disease-dependent manner. This panel was aimed, mainly, at the detection of DNA bacteriophages specific for *Enterobacterales* and some human viral pathogens [28].

### 4.4. Possible Interaction Between E. coli and Bacteriophages

We have found relatively high contents of *E. coli* post-FMT in GVHD patients, along with low abundance of *E. coli*-hosted phages. Meanwhile, an increased abundance of *Enterobacteria* phages (especially, *Eganvirus*) was seen in Crohn’s disease after FMT. A reverse correlation between *E. coli* and *Eganvirus* contents in GVHD patients suggests a possible bacteriolytic effect of these, generally, temperate, *Enterobacteria* phages. *Eganviruses* comprise a group of 186-type-like bacteriophages infecting different *Enterobacteria* [52]. The *Eganviruses* infect, mainly, *E. coli* and related *Enterobacterales*. Its abundance post-FMT showed negative correlation to relative contents of *E. coli*, their presumable host. Sufficient increase in *Eganvirus* species in Crohn’s disease at 30 days after FMT may reflect their significance as potentially lytic phages for *E. coli* regulation. Antibacterial effects of other bacteriophage species and their occasional associations with different *Enterobacteria* requires further studies in terms of bacterial host specificity.

Hence, modification of the phage landscape following FMT may be of both diagnostic and therapeutic use in GVHD and Crohn’s disease [53]. To this point, one should perform special studies on the transfer of virome components from healthy donors to the FMT recipients. The first study of such kind was presented by Ott et al. [54]. The authors have reported good clinical effect with transplantation of sterile fecal filtrate to the patients with *Clostridium difficile* infection. Potential positive effects produced by the phage component of fecal microbiota transfer were discussed in a recent review article [18]. Still, some issues of FMT safety and standardization remain open in absence of large randomized studies in HSCT and GVHD patients. To make the results of FMT more reproducible and comprehensive, some authors propose separate transplantation of viral or microbial fractions of fecal transplant in order to compare the efficiency of distinct microbiome components [17].

Our study has some limitations: (1) NGS data for donor fecal microbiota are lacking, thus allowing only preliminary conclusions on the origin of bacteriome changes. (2) Gut barrier damage should be characterized with broader set of quantitative biomarkers [55], including plasma endotoxin levels, circulating bacterial and viral DNAs, in order to assess the degree of intestinal wall lesions in distinct disorders. (3) Another limitation of our study was lack of GVHD-specific biomarkers (ST2 and REG3α) predicting clinical outcomes according to the guidelines issued by the Mount Sinai Consortium [41].

## 5. Conclusions

FMT was applied as a novel treatment option for microbiota reconstitution in severe intestinal GVHD and Crohn’s disease. These immune-mediated disorders still have different clinical and pathological patterns. In both groups of patients, gut microbiota was sufficiently affected by previous antibiotic treatment. Before FMT, the relative contents of anaerobic bacteria were disturbed in both intestinal GVHD and Crohn’s disease versus the control group. The intestinal cell damage, being evaluated by low plasma citrulline levels, was more pronounced in GVHD than in Crohn’s disease, being associated with increased fecal *Klebsiella*, *E. coli*, and *Enterococcus* abundances after FMT. Meanwhile, the increased *Faecalibacteria, Romboutsia,* and *Dialister* ratios were found in Crohn’s disease versus GVHD at different timepoints after FMT, thus suggesting lesser damage to anaerobic microbiota in Crohn’s disease. A customized NGS panel detected a transient increase in *E. coli*-hosted phages in Crohn’s disease post-FMT. In GVHD patients, a negative correlation was revealed between *E. coli* and *E. coli*-hosted *Eganvirus* species, thus suggesting a significant role of *Eganvirus* for clinical effect of FMT in GVHD patients. Clinical response to FMT in GVHD patients was shown to correlate with increased plasma citrulline. Future studies should specify the relationships between fecal bacteriome and virome reconstitution following FMT in gut GVHD and other immune-mediated intestinal disorders.

## Figures and Tables

**Table 1:** Pre-FMT abundance of most common bacterial genera in fecal samples of GVHD patients and in Crohn’s disease assessed by 16S rRNA NGS (percent values, M ± m, presence of >0.5%).

Microbial Genera	GVHD (n = 12)	Crohn’s Disease (CD), n = 15	Control Group (n = 148)	GVHD vs. CD, *p* Values	GVHD vs. Control Group, *p* Values	CD vs. Control Group, *p* Values
1	2	3	4	5	6	7
*Akkermansia*	0.90 ± 0.85	15.03 ± 6.52	2.11 ± 0.41	0.090	0.03	0.557
*Alistipes*	0.03 ± 0.02	0.43 ± 0.23	3.17 ± 0.23	0.50	<0.001	<0.001
*Bacteroides*	2.77 ± 1.77	2.04 ± 1.24	23.79 ± 1.07	0.497	<0.001	<0.001
*Bifidobacterium*	0.07 ± 0.04	8.41 ± 5.24	1.95 ± 0.27	0.044	<0.001	0.130
*Blautia*	0.19 ± 0.16	0.13 ± 0.10	0.69 ± 0.07	0.792	<0.001	<0.001
*Clostridium XIVa*	3.71 ± 3.36	0.27 ± 0.14	0.08 ± 0.02	0.649	0.775	0.832
*Collinsella*	1.28 ± 1.27	0.71 ± 0.71	0.78 ± 0.10	0.820	<0.001	<0.001
*Desulfovibrio*	0.00	1.67 ± 1.66	0.16 ± 0.06	0.180	0.021	0.193
*Dialister*	0.10 ± 0.07	3.75 ± 1.91	1.53 ± 0.20	0.026	0.007	0.451
*Dorea*	0.12 ± 0.11	0.60 ± 0.39	0.48 ± 0.05	0.261	<0.001	0.017
*Enterobacter*	3.040 ± 3.04	0.002 ± 0.002	0.110 ± 0.073	0.877	0.363	0.233
*Enterococcus*	8.560 ± 4.88	0.07 ± 0.06	0.040 ± 0.014	0.007	<0.001	0.739
*E. coli*/*Shigella*	12.04 ± 5.97	5.29 ± 2.94	0.63 ± 0.12	0.722	0.535	0.561
*Faecalibacterium*	3.00 ± 2.25	16.92 ± 6.21	6.09 ± 0.29	0.203	<0.001	0.764
*Fusobacterium*	1.43 ± 1.41	4.76 ± 2.73	0.18 ± 0.13	0.331	0.255	0.003
*Haemophilus*	1.16 ± 1.15	0.12 ± 0.11	0.16 ± 0.06	0.862	0.208	0.144
*Klebsiella*	30.19 ± 9.54	12.10 ± 6.75	0.12 ± 0.05	0.285	<0.001	0.001
*Lachnospiracea*	2.55 ± 1.78	0.47 ± 0.26	0.12 ± 0.05	0.415	0.035	<0.001
*Lactobacillus*	0.06 ± 0.03	1.86 ± 1.66	0.22 ± 0.16	0.412	0.028	<0.001
*Parabacteroides*	1.28 ± 1.12	2.09 ± 1.95	2.53 ± 0.18	0.880	<0.001	<0.001
*Phascolarctobacter*	0.02 ± 0.02	0.01 ± 0.01	2.57 ± 0.23	0.893	<0.001	<0.001
*Prevotella*	0.06 ± 0.02	4.18 ± 2.66	7.28 ± 0.91	0.686	0.004	0.148
*Romboutsia*	0.40 ± 0.40	0.84 ± 0.66	0.10 ± 0.02	0.175	0.002	0.276
*Roseburia*	0.13 ± 0.09	0.33 ± 0.19	1.19 ± 0.12	0.331	<0.001	<0.001
*Ruminococcus*	0.02 ± 0.01	0.71 ± 0.68	1.73 ± 0.21	0.284	<0.001	<0.001
*Staphylococcus*	2.90 ± 2.90	0.01 ± 0.004	0.005 ± 0.004	0.879	0.008	0.165
*Sutterella*	0.01 ± 0.01	0.31 ± 0.31	1.08 ± 0.11	0.959	<0.001	<0.001
*Veillonella*	3.17 ± 1.65	4.95 ± 2.50	0.09 ± 0.03	0.701	0.090	0.489

Note: Intergroup differences significant at *p* < 0.05 are shown in bold letters.

**Table 2:** Relative pre-FMT abundance of common viral genes (>0.5% of total fecal DNA) detected by a customized NGS panel (M ± m).

Viral Genes: Classes	GVHD (% of Total DNA)	Crohn’s Disease (% of Total DNA)	*p* Values
*Caudoviricetes*	86.59 + 6.19	87.09 + 7.83	0.524
*Herviviricetes*	0.37 + 0.37	4.96 + 3.87	0.728
*Megaviricetes*	1.51 + 1.15	0.39 + 0.32	0.164
*Pokkesviricetes*	8.89 + 4.16	7.45 + 6.58	0.236
*Tectiliviricetes*	0.00 + 0.00	0.026 + 0.025	0.143
Viral genes: Genera			
*Bievrevirus*	1.91 + 1.87	6.17 + 5.26	0.197
*Brunovirus*	2.01 + 1.16	0.39 + 0.34	0.411
*Carjivirus*	0.11 + 0.11	0.68 + 0.59	0.330
*Cytomegalovirus*	0.37+ 0.37	4.96 + 3.78	0.728
*Eganvirus*	16.98 + 14.05	17.73 + 11.33	0.854
*Irrigatiovirus*	1.73 + 1.73	0.068 + 0.058	0.944
*Jouyvirus*	0.02 + 0.02	0.35 + 0.35	0.804
*Lambdavirus*	1.07 + 0.68	12.33 + 7.91	0.433
*Lederbergvirus*	27.17 + 17.36	31.73 + 11.70	0.586
*Lymphoc dis virus*	1.51 + 1.15	0.39 + 0.32	0.164
*Mastadenovirus*	0.00 + 0.00	0.026 + 0.023	0.143
*Novemvirus*	1.74 + 1.74	2.98 + 2.57	0.576
*Oryzopoxvirus*	8.89 + 4.16	7.45 + 6.58	0.236
*Senquatrovirus*	0.037 + 0.037	0.089 + 0.065	0.576
*Shamshuipovirus*	0.042 + 0.042	0.077 + 0.052	0.340
*Tequatrovirus*	4.41 + 3.18	3.36 + 3.17	0.553
*Traversvirus*	0.19 + 0.09	0.79 + 0.24	0.026
*Wanchaivirus*	0.11 + 0.10	0.37 + 0.29	0.503

**Table 3:** Average values of fecal virome biodiversity before FMT in GVHD and Crohn’s disease patients (M + m).

Biodiversity Indexes	GVHD	Crohn’s Disease	*p* Values
Shannon index	1.45 + 0.35	1.10 + 0.15	0.615
Simpson index	0.60 + 0.10	0.49 + 0.07	0.366
Chao 1	25.26 + 7.26	13.57 + 1.80	0.085
Se_chao 1	7.51 + 1.81	3.32 + 0.87	0.111

**Table 4:** Correlations between relative contents of *Escherichia*/*Shigella*, and *E. coli*-hosted phages in GVHD (41 pairs) and Crohn’s disease groups (55 pairs).

	GVHD, All Time Points (n = 41)		Crohn’s Disease, All Time Points (n = 55)	
	r	*p*	r	*p*
*Eganvirus EtG*	−0.521	2.4 × 10^−4^	−0.018	0.450
*Eganvirus ev186*	−0.527	2.0 × 10^−4^	−0.018	0.450
*Eganvirus PsP3*	−0.535	1.6 × 10^−4^	0.073	0.298
*Eganvirus SEN1*	−0.511	3.2 × 10^−4^	−0.106	0.221
*Salmonella phage BIS20*	−0.553	9.0 × 10^−5^	0.063	0.324

Note: correlations are considered significant at *p* < 0.001.

## Data Availability

The original contributions presented in this study are included in the article/Supplementary Material. Further inquiries can be directed to the corresponding author.

## Supplementary Materials

The following supporting information can be downloaded at: https://www.mdpi.com/article/10.3390/microorganisms13102337/s1, Supplemental Table S1. Clinical characteristics of GVHD patients, Supplemental Table S2. Clinical characteristics of patients with Crohn’s disease (percentage of cases shown in parentheses).
