# Supplementary material for: Changes in Gut Phageome and Bacteriome Following Fecal Microbiota Transfer in Patients with Intestinal Graft-Versus-Host Disease and Crohn’s Disease
Source: Microorganisms. 2025 Oct 10;13(10):2337. doi: 10.3390/microorganisms13102337 (PMC12566361; doi:10.3390/microorganisms13102337)
Supplement: Supplementary file 1 [file microorganisms-13-02337-s001.zip › microorganisms-3841500-supplementary.pdf]

Supplementary Table S1. Clinical characteristics of GVHD patients.

**Demography:**	n=12
Median age, years (range)	32(9-53)
Males/females	.7/5 (58/42%)
**Primary diagnosis, number of cases (% of total)** :	12 (100%)
Acute lymphoid leukemia	2(16)
Acute myeloid leukemia	3(25)
Myelodysplastic syndrome	1(8)
Chronic myeloid leukemia	4(33)
Aplastic anemia	1(8)
Fanconi anemia	1(8)
**Remission prior to HCT, n(%):**	
Yes	8(67)
No	4(33)
**HSCT donor type, n(%):**	
Allogeneic unrelated	10(83)
Haploidentical	2(17)
**Conditioning regimen, n(%):**	
FluBu12	1(8)
FluBu10	8(67)
FluBu8	1(8)
FluBu4ATG	1(8)
CyFluATG	1(8)
**GVHD prophylaxis n(%):**	
CyTxMMF	8(67)
CyCyAMMF	1(8)
CyCirRuxo	1(8)
CyTxMMFBendamustin	1(8)
CyTxRuxo	1(8)
**GVHD grade, n(%):**	12(100)
Overall GVHD grade	
4	10
3	2
Acute skin GVHD, grade:	
1	1(8)
2	4(33)
3	1(8)
Acute GVHD, intestinal, grade	
3	8(67)
Acute hepatic GVHD, grade	
2	2(16)
Overlap acute/chronic GVHD (skin), grade, n(%)	
1	1(8)
2	1(8)
Overlap acute/chronic GVHD (intestinal)	
2	2(16)
3	2(16)
Overlap hepatic GVHD	

3	1(8)
Overlap acute/chronic GVHD, mucosal, grade	
1	3(25)
**Conventional GVHD therapy, n (%):**	
Glucocorticosteroids	1(8)
Sirolimus+Steroids	1(8)
Tacrolimus+Steroids+Ruxolitinib	7(58)
Tacrolimus +Steroids+Ruxolitinib+Anti-TNF	2(16)
Tacrolimus +Steroids+Ruxolitinib+Photopheresis	1(8)
**Indications for FMT, n(%):**	
Acute intestinal GVHD	8(67)
Overlap acute/chronic intestinal GVHD	4(33)
**Intestinal coinfections in GVHD patients, n (%):**	
Cl. Difficile	2(16)
Viral pathogens, case number (% of total):	8(67)
CMV	6(50)
HHV-6	9(75)
EBV	2(17)
K.pneumonia	5(42)
**Time period from HCT to FMT, days:**	108.5(41-585)
Time from GVHD detection to FMT, days	57.5(23-313)
**Clinical response after FMT:**	
Complete response	6(50)
Partial response	4(33)
No detectable response	2(16)
***C.difficile* eradication post-FMT, d+15, n(%)**	2(100)
K.pneumonia decolonization:	
Early decolonization of *K.pneumonia,* d+30	1(20)
Decolonization of *K.pneumonia* d+60	2(40)
Absence of *K.pneumoniae* decolonization	2(40)
**Serious adverse effects post-FMT**	0

Supplementary Table S2. Clinical characteristics of patients with Crohn's disease (percentage of cases shown in parentheses).

**Disease phenotype, No. of cases (% of total)**	
B1 inflammatory	12(80)
B2 stricturing	1(7)
B3 penetrating	2(13)
Intestinal sites affected, Number of cases (% of total)	
L2 colitis	8(53)
L3 ileocolitis	7(47)
Clinical activity before FMT (Harvey-Bradshaw index)	
Remission	2(13)
Low activity	5(33)
Moderate activity	5(33)
High activity	3(20)
Pre-FMT complications	
Abdominal infiltrate	1(7)
Strictures	2(13)
Intestinal obstruction	1(7)

Pararectal fistules	2(13)
Fistules at other sites (stomach, bronchial etc.)	1(7)
Liver cirrhosis	1(7)
Anal fissures	2(13)
Pre-FMT surgery	
Right-side colectomy	1(7)
Small intestine resection	2(13)
Anal fissure excision	1(7)
Draining ligature	2(13)
Appendectomy	1(7)
Orthotopic liver transplantation	1(7)
Previous drug therapy	
5-aminosalicylates, n(%)	12(80)
Glucocorticosteroids, n(%)	14(93)
Cytostatics, n(%)	12(80)
Anti-TNF drugs, n(%)	5(33)
Other immune modulators, n(%)	15(100)
Other immune suppressors, n(%)	1(7)
Pre-FMT treatment of *C. difficile* infection	
Vancomycin therapy, oral	13(87)
Vancomycin therapy, rectal	6(40)
Metronidazole therapy	10(67)
C.difficile eradication (day +15 after FMT), total (%)	14 (93)
Clinical activity of Crohn's disease on day +60 after FMT (Harvey-Bradshaw index)	
Remission	10(67)
Low activity	4(27)
Moderate activity	0
High activity	1(7)
